# “Are you safe to talk?”: Perspectives of Service Providers on Experiences of Domestic Violence During the COVID-19 Pandemic

**DOI:** 10.1007/s10896-022-00359-9

**Published:** 2022-01-14

**Authors:** Jenny K. Leigh, Lita Danielle Peña, Ashri Anurudran, Anant Pai

**Affiliations:** 1 Present Address: COVID-19 Task Force On Domestic Violence, 1629 K Street, NW #300, Washington DC, 20006 USA; 2 Department of Sociology, New York University, New York, NY USA; 3 Harvard Medical School, Boston, MA USA; 4 Institute of Psychiatry, Psychology, & Neuroscience, King’s College London, London, UK

**Keywords:** Domestic violence, COVID-19, Coronavirus, Stay-at-home, Shelter-in-place, Service providers

## Abstract

This study aimed to better understand the factors driving reported trends in domestic violence during the COVID-19 pandemic, particularly the effect of the pandemic on survivors’ experiences of violence and ability to seek support. We conducted semi-structured qualitative interviews with 32 DV service providers operating in organizations across 24 U.S. cities. The majority of providers described a decrease in contact volume when shelter-in-place orders were first established, which they attributed to safety concerns, competing survival priorities, and miscommunication about what resources were available. For most organizations, this decrease was followed by an increase in contacts after the lifting of shelter-in-place orders, often surpassing typical contact counts from the pre-pandemic period. Providers identified survivors’ ability to return to some aspects of their pre-pandemic lives, increased stress levels, and increased lethality of cases as key factors driving this increase. In addition, providers described several unique challenges faced by DV survivors during the pandemic, such as the use of the virus as an additional tool for control by abusers and an exacerbated lack of social support. These findings provide insight into the lived experiences driving observed trends in DV rates during COVID-19. Understanding the impact of the pandemic on survivors can help to shape public health and policy interventions to better support this vulnerable population during future crises.

In response to the onset of the COVID-19 pandemic, governments across the world introduced large-scale mitigation efforts, including “stay-at-home” or “shelter-in-place” mandates. These mandates attempted to protect residents from the coronavirus and reduce its spread by keeping people at home and shutting down non-essential businesses. However, amidst these efforts to protect public health, the vulnerabilities of some at-risk populations have been heightened (Kofman & Garfin, 2020). For those experiencing domestic violence (DV) during this time, home may have become less safe than ever.

Domestic violence affects about 1 in 3 women and 1 in 7 men in the United States (Smith et al., 2018). For the purposes of this study, we refer to domestic violence as it pertains to partners in a relationship (also often referred to as intimate partner violence, relationship violence, or dating abuse in the literature). The consequences of DV for survivors, their families, and society have contributed to the growing global recognition of DV as a pervasive public health burden (Garcia-Moreno & Watts, 2011). Violence within the home, whether it be physical, sexual, or psychological, can contribute to a myriad of negative health effects, including immediate trauma, unwanted pregnancy, unsafe abortions, sexually transmitted infections, depression, and anxiety (World Health Organization, 2005). Furthermore, childhood exposure to domestic violence, even as a witness, increases the risk of growth stunting, behavioral health disorders, and distrust of caregivers (Pernebo & Almqvist, 2017). In addition to these health consequences, DV can also contribute to diminished economic productivity, as well as increased medical and judicial costs for the community in which the violence takes place (Pearl, 2013; Peterson et al., 2018).

Importantly, DV has been shown to increase under conditions of stress (Cano & Vivian, 2001), social isolation (Coohey, 2007), and economic hardship (Anderberg et al., 2016), factors which are often present during major catastrophes, including the current COVID-19 crisis. As such, researchers and policymakers alike anticipated a surge in domestic violence cases both in the United States and across the world during this time, in addition to even greater barriers to survivor help-seeking (Bradbury-Jones & Isham, 2020; Kofman & Garfin, 2020). Recent studies have indeed revealed increases in rates of domestic violence in both the U.S. and globally, with some referring to this co-occurrence as a “double pandemic” (Bettinger-Lopez & Bro, 2020). However, empirical work examining the lived experiences shaping these observed changes remains limited.

What underlying factors drove the reported trends in domestic violence during the early months of the COVID-19 pandemic, and how did new obstacles influence survivors’ ability to seek support? Such research is crucial to determine how public health interventions and government policies can best support survivors during future disasters where similar measures might be necessary. This study addresses this need by interviewing 32 domestic violence service providers in 24 U.S. cities about the challenges and barriers to support that survivors faced during the COVID-19 pandemic.

## Literature Review

Prior to the COVID-19 pandemic, both academic scholarship and research by national anti-violence organizations helped to advance our understanding of how disasters influence experiences of gender-based violence. Studies of disasters such as the Deepwater Horizon oil spill, Hurricane Katrina, and the 2010 earthquake in Haiti found that the likelihood of experiencing domestic violence increased dramatically for directly-impacted survivors (Buttell & Carney, 2009; Lauve-Moon & Ferreira, 2017; Schumacher et al., 2010). A recent study of survivors’ and agencies’ experiences during Hurricane Harvey further underscored the increased vulnerability of survivors, including their lack of both material and support resources (Serrata & Hurtado Alvarado, 2019). Disasters often cut survivors off from their social support networks, increase feelings of stress, and threaten their economic stability, all of which impede survivors’ ability to respond to violence (Sety et al., 2014). 

However, the COVID-19 pandemic raises unique concerns compared to these previously-studied events (Usher et al., 2021). Particularly during the early months of the pandemic, the lack of knowledge about what exactly the novel coronavirus was, uncertainty around how to best protect oneself, and extended periods of social isolation posed distinct psychological and social challenges. This study thus adds to this growing body of literature by offering insight into several factors driving changing rates of domestic violence during the pandemic, as well as identifying some of the specific difficulties that survivors confronted.

Over the past year, a growing number of academic reports have discussed the potential effects of the COVID-19 pandemic on domestic violence, with many articles speculating an increase in incidence rates, as observed during previous disasters (e.g. Bradbury-Jones & Isham, 2020; Ertan et al., 2020; Kofman & Garfin, 2020; Malathesh et al., 2020; Mazza et al., 2020). Empirical research on the topic has supported these predictions. In one of the earliest analyses during COVID-19, Boserup et al. (2020) drew attention to spikes in DV-related police calls in various U.S. cities. For example, their analysis of data from the San Antonio Police Department revealed an 18% increase in calls pertaining to family violence in March 2020 (shortly after stay-at-home orders were established in the county) compared to March 2019. Similar trends were seen after stay-at-home orders were implemented in Oregon, Alabama, New York, as well as Georgia (Evans et al., 2020).

Studies from other countries have also observed rising reports of domestic violence. A study by Ravindran and Shah (2020) conducted in India similarly found an increase in domestic violence and cybercrime complaints after shelter-in-place orders were initiated, with increases most concentrated in districts with the strictest lockdown orders. In Australia, Boxall and colleagues (2020) sent an online survey to 15,000 women about their experiences of domestic violence during the initial three months of the COVID-19 pandemic. Results suggested that the pandemic coincided with the onset or escalation of violence and abuse among many Australian women. A recent systematic review and meta-analysis by Piquero et al. (2021) examined data from 18 international studies and found a moderate to strong increase in domestic violence between pre- and post-lockdown periods. However, the authors noted that “the exact nature and context of the increase remains unknown” (p. 7). While these studies have shown notable shifts in rates of DV during the pandemic, additional empirical research is needed to explain what factors might have driven these trends.

Moreover, despite growing insight into changes in DV during the pandemic, researchers have also pointed to inconsistencies in reported trends depending on how domestic violence is measured (i.e., domestic crimes, arrests for domestic crimes, calls to hotlines) and who is reporting the data (i.e., service providers vs. law enforcement). For example, one paper that utilized a variety of data sources found that though calls to both a local hotline and law enforcement increased during the shelter-in-place period, domestic crimes and associated arrests for these crimes decreased (Miller et al., 2020). In addition to variation across different measures of DV, these trends further shifted looking at different points in time, with decreases in all measures reported after the start of re-opening. The impact of temporal scope was also reflected in a recent time-series analysis of police data in Dallas, which indicated an initial spike in Family Violence Incident reports in the first two weeks of lockdown, followed by a decrease in the next three weeks (Piquero et al., 2020).

These limitations of quantitative findings indicate the need for in-depth qualitative research to better understand the social context in which observed trends occur, as well as what might be driving the reported increases and decreases in these measures of domestic violence over time. A few qualitative studies have already helped to address this gap in our understanding of experiences of DV during COVID. Other qualitative studies examining perspectives of survivors and families have been published in India (Huq et al., 2021), Greece (Chatzifotiou & Andreadou, 2021), and England (Gregory & Williamson, 2021). Recent work by van Gelder et al. (2021) examined the increasing professional and personal challenges that providers in the Netherlands experienced while supporting survivors remotely during the first wave of the pandemic. As providers interact with a large number of survivors and address a variety of experiences, their perspective can provide insight into the common factors shaping broader trends related to DV. However, no qualitative studies exploring providers’ understanding of changes in DV during COVID-19 have been conducted in the U.S. to date. This study will address this important gap in the literature by examining the changes in experiences of domestic violence that service providers observed through their work with survivors during the pandemic.

## Methods

To understand the factors shaping reported trends in domestic violence during the pandemic, we conducted in-depth interviews with 32 domestic violence support providers in 24 U.S. cities.

### Sample

Domestic violence service providers were chosen as interview participants due to their direct access to and experience with survivors. Service providers are able to address broader trends seen across their many clients, as well as share insight on changes seen across the pre-pandemic and pandemic periods. Crucially, speaking to domestic violence service providers does not risk the safety of survivors themselves. In order to incorporate multiple perspectives, we spoke with service providers working in a variety of roles at their agencies, from Advocates to Executive Directors. We also drew from various services within organizations, including clinical services, residential and housing services, hotlines, and legal support, among others.

We chose not to conduct interviews with law enforcement officers, another population who may have contact with domestic violence survivors, for a variety of reasons. First, only a small portion of domestic violence incidents are reported to the police (Dziegielewski & Swartz, 2007). Additionally, domestic violence includes not only physical and sexual violence (which is often what leads to police being called to the home), but also other forms of power and control perhaps not visible to law enforcement officers, particularly those with little to no domestic violence-related training. As such, police officers may not be able to provide a nuanced account of the experiences of survivors during this time. Further, community relations with law enforcement officers are particularly strained in the current social and political climate (Cole et al., 2020). Even prior to the growing attention to racial injustice in the past year, people of color and other marginalized groups have described facing additional barriers to reporting, including safety concerns when engaging with police (Calton et al., 2016; Decker et al., 2019; Braga et al., 2019).

Of the 32 organizations interviewed, 11 were located in the West, 6 in the Midwest, 8 in the South, and 7 in the Northeast. Organizations operated at the county-level, primarily servicing women, and often low-income women of color in particular. A few organizations serviced specific populations, such as LGBTQ + survivors, immigrants, or survivors with disabilities. The providers interviewed included 28 women and 1 genderqueer person. 16 identified as White, 5 identified as multi-racial, 4 identified as Latinx, 3 identified as Asian, and 1 identified as African-American. 3 providers chose not to disclose demographic information. The number of years spent in domestic violence-related work ranged from 2 to 30, with an average of 11 years; the amount of time at their respective agencies ranged from 6 months to 27 years, with an average of 9 years.

### Recruitment

Domestic violence support organizations were identified using the aggregator platform domesticshelters.org, which lists support providers by zip code. We aimed to identify organizations in approximately 30 major U.S. cities and their surrounding areas within all four major census regions. This would allow us to better understand the conditions which may contribute to previously observed quantitative trends. We reached out to potential organizations via email, explaining our research aims and requesting a 30-min remote interview with a service provider in their organization. Organizations were also provided with a copy of the interview questions in the initial outreach email.

### Interviews

Semi-structured interviews were conducted remotely between July and November of 2020 via phone or the video calling platform Zoom. Trained members of the investigative team followed an interview guide with a set list of questions determined by the team. A semi-structured interview approach was chosen as it establishes consistency across interviews while also allowing for follow-up questions and a more natural flow of conversation (Longhurst, 2003). Key interview questions can be found in Table 1.

**Table 1:** Key interview questions from interview guide

*How are people experiencing domestic violence reaching out to you? Is this any different from what it was like before the pandemic? What do you think might be motivating these differences?*
*What factors have you seen that influence the ability of a person to reach out for help during this time?*
*Have domestic violence contacts increased, decreased, or remained the same over the past several months? What do you think might be driving these trends?*
*When people reach out to you, how do they describe the situations they are experiencing? Is this any different from what the situations were like before the pandemic? What do you think might be motivating these differences?*

Interviewers took typed notes during the course of each interview. Interview audio was also recorded upon permission by the domestic violence service providers to facilitate the transcription of direct quotes.

### Analysis

The interview text was coded using an iterative approach (Srivastava & Hopwood, 2009). An iterative approach combines elements from both deductive (i.e., key codes created on a priori basis and serve as a template for data analysis) and inductive (i.e., key codes created on the researcher’s interpretation of the text) analysis. In this study, a template was developed from a priori codes but could be changed (e.g., add new codes, combine codes) as analysis continued. Data were analyzed thematically. Thematic analysis is a method for identifying, analyzing, and reporting patterns of meaning within qualitative data. Thematic analysis was chosen as it allows theoretical flexibility (Braun & Clarke, 2006).

### Ethical Considerations

The study protocol was submitted to the Columbia University Institutional Review Board and determined to pose no more than minimal risk to study participants. When directly quoting providers, we refer solely to the region in which they worked at the time of the interview, avoiding the names of individuals or individual organizations.

## Results

When describing how, when, and why survivors reached out, most providers’ observations converged around one of two temporal patterns. The most common trend involved a substantial decrease in the volume of contacts to providers when shelter-in-place orders were first established, followed by an ongoing increase in volume as shelter-in-place orders were lifted. In most cases, providers reported that contacts had actually surpassed normal levels in the months after shelter-in-place orders began to be lifted. The second most common trend was simply a consistently high – in many cases, above-average – volume of contacts beginning at the start of shelter-in-place orders, which in some cases continued to increase as the pandemic unfolded.

Before analyzing these trends in more depth, it is necessary to clarify some common descriptors. Because we spoke with providers in a variety of states, the timing of the enforcement and lifting of shelter-in-place orders did not align across agencies. Because of this state-by-state variation, we avoid referring to specific months in our descriptions of trends, and instead refer to “the start of shelter-in-place orders” and “the lifting of shelter-in-place orders” as key markers of time. Because we conducted interviews during the first wave of COVID-19, the start of shelter-in-place orders corresponds to roughly mid-March of 2020 in most regions, and the lifting of shelter-in-place orders corresponds to late spring or early summer, depending on the state. Additionally, as agencies use a variety of platforms to communicate with survivors, we refer to all forms of communication with survivors – whether via phone, chat, or text – as “contacts,” and specify the platform when relevant. We largely avoid the use of “reports” to refer to these communications, as this language tends to connote formal reporting.

### Factors Influencing a Decrease in DV Contacts

The majority of providers described an initial decrease in the volume of contacts they received during the early weeks of shelter-in-place orders. Providers emphasized that this decrease was notably not because domestic violence itself was decreasing during these early weeks, but because survivors were facing more complex barriers to outreach in the climate of the pandemic. “It was eerie,” one provider in Texas said. “We know that DV is not over. We know that now is a higher-stress time – it’s most likely increasing. But we just weren’t getting calls.” Their knowledge that domestic violence was certainly still occurring was later reinforced by their communications with survivors who had not been able to reach out during the peak of the pandemic. To explain the low contact volume during the first several weeks of shelter-in-place orders, providers identified several common barriers to access, including survivors’ safety concerns, their need to balance competing survival priorities, and a lack of clear communication about what resources were available.

#### Safety Concerns

By far, the most common reason that providers gave to explain the initial decrease in contacts was the challenge of how to reach out safely during a time when most survivors were forced to be with their abusers 24/7. While in normal times, survivors might have been able to rely on leaving the house to go to work, run errands, or meet up with friends – valuable moments of freedom when they might have been able to reach out – the pandemic made it impossible for survivors to find a moment alone. When describing the conditions that many survivors faced as a result of shelter-in-place orders, one provider in Minnesota said, “When your home isn’t safe and you need to leave but you’re stuck there, how are you gonna call and tell someone, ‘Hey, my home isn’t safe,’ when the person who’s making your home unsafe is standing right next to you?” Providers also reflected that when survivors were able to find some time and space away from their abuser, conversations were often rushed, even ending abruptly at times. This changed the dynamic of many conversations, as another provider in Texas described:

Before the pandemic, when people would reach out to us, we could have a… not a lengthy conversation, but there wasn’t really this hurried approach to it. We could ask follow-up questions if you said something that indicated that there was a little bit more underneath that… And now it seems that we don’t have the space for that all the time, so we have to be pretty quick and abrupt at some level.

With awareness of their limited time, providers described shifting to prioritize immediate safety needs even more than they normally would, which in many cases prevented them from exploring survivors’ situations as fully as they might have wanted. Shelter-in-place orders thus may have helped to keep the coronavirus out of survivors’ homes, but left them vulnerable to a different kind of danger, with fewer options for support.

#### Competing Priorities

In addition to safety concerns, providers also pointed to the reality that for many survivors, the violence they were experiencing may not have been their biggest concern during such a tumultuous time. Such observations underscore the severity of other social and economic challenges survivors were facing, including losing their jobs and homes, figuring out how to get basic necessities, having to support children at home, and coping with the death of loved ones. Describing how survivors were forced to go into even deeper “survival mode,” one provider said, “Going back into Maslow’s hierarchy of needs, the domestic violence wasn’t the biggest issue any more, or getting out, it was ‘How do I stay safe from COVID?  How do I protect my kids? I can’t even talk about leaving right now, I’m just in pure survival mode right now.’” With more immediate concerns to address, survivors were not able to prioritize leaving a violent situation, a concerning reflection of the numerous social consequences of the pandemic. Another provider in Florida explained:

When people are living in times of mass tragedy, like 9/11, a pandemic, with so much uncertainty in their lives in general, I feel like… part of the reason they’re not necessarily reaching out for help is because while the domestic violence is not good, getting up and running away to an unknown shelter or place or turning their life additionally upside down is overwhelming.

A provider in California similarly likened the effects of the pandemic to other major tragedies her clients had been coping with, such as the wildfires that had been unfolding concurrently. “For humans in general, it’s almost like you have enough to deal with and you can’t add one more change – one major change – to your life… That’s how I would explain it, because we’ve seen it before. The same thing happened during the crisis with the earthquake [in 2018] and the fires.” Understandably, during a moment of crisis, survivors would want to hang on to the limited stability that they have, even if it poses its own dangers.

#### Lack of Clarity on Available Resources

Compounding the existing difficulties survivors often face in accessing information about DV resources, the lack of clear communication about what resources were still open during the pandemic also contributed to the initial decrease in call volume. One service provider from New York City described, “People thought that since we weren’t seeing people in person, that the program closed and we weren’t taking intakes or referrals. So I think there was just a lot of miscommunication when all of this started, and people didn’t understand what was happening.*”* A service provider in Oregon similarly emphasized the lack of clarity: “It was so confusing, for even us in the field – I can imagine it was 20 times more confusing for survivors.” Though organizations took steps to communicate that they were indeed providing services for survivors, such efforts did not have an effect until several weeks into the pandemic, as described in the next section.

### Factors Influencing an Increase in DV Contacts

Nearly all service providers described an increase in domestic violence contacts during the pandemic. This increase followed a sharp initial decrease during the first few weeks of the pandemic for the majority of organizations. In many cases, the subsequent increase in domestic violence contacts surpassed the typical number of contacts that providers would expect in the pre-pandemic period. Some key factors that providers recognized in explaining this increase included survivors’ ability to return to activities from their pre-pandemic lives, a greater need for resources that support organizations could offer, heightened feelings of stress, and higher lethality of violence.

#### Return to Normality

One key explanation as to why providers observed an increase in contacts after the first several weeks of the pandemic was because many survivors were able to begin resuming some aspects of their normal lives again. For some agencies, the increase associated with this return to semi-normalcy brought them back up to their usual contact volume, as a provider in Texas recalled: “When a lot of the restrictions were lifted and shelter-in-place ended, it’s like the switch turned on. We went from just a few calls to back to normal, like, forget that there’s COVID happening.” Reflecting on reasons for the increase that she had observed after the initial decline in calls, a provider in Missouri explained, “As it’s stretched on, we’ve just seen more people call needing help, whether that be some things are opening up again, some people are getting jobs again, you know, having opportunities to slip away or having those opportunities to call.” As survivors were able to leave their house more often or perhaps return to work, they were able to once again create opportunities to reach out for help.

In response to circumstances that made outreach more difficult for survivors, many agencies worked to quickly create or expand chat or text services. Because chat and text platforms can facilitate communication more discreetly than making a phone call would, some providers reported an increase in contacts via these channels, as well as e-mail. “The chat function now allows people to communicate with us even when their abuser is in the room,” one advocate in Minnesota described. Some agencies with existing chat and text services extended their hours, while those who did not have them began to create them. While providers may not have been able to change survivors’ home environments, many were able to rapidly respond to shifting circumstances and enable survivors to reach out in new ways.

Additionally, in response to the lack of clarity around which resources were still accessible during the pandemic, many organizations introduced creative strategies to raise awareness about their ongoing services and inform survivors that their services remained available. A Florida provider explained:

We spent a lot of time connecting with local media, social media, any organizations that were holding virtual town halls, and we hosted our own, trying to do live on Facebook, and trying to get the message out that we are still open. You can still call us, we are still taking people into shelter, we have safety measures in place.

As shelter-in-place orders were lifted, survivors also became more aware of available support services, offering another explanation for the increase in contact volume described by providers. A provider from New York City further elaborated on this change in survivors’ awareness of resources:

Maybe because people understand that we are here, versus in the beginning of the pandemic, there was a lot of confusion if shelters were taking people, if community services were open and serving. So it might just be access and understanding that okay, they are there, just in this format, in a different way, and I am going to reach out for help.

However, while the lifting of shelter-in-place orders and increased awareness of available resources may explain why contacts increased to normal levels, they do not explain why contacts would have *exceeded* them, as was the case for many agencies.

#### Requests for Resources

One potential explanation providers offered for the above-average volume of contacts was the increased demand for housing, food, childcare, and other survival necessities during the pandemic. As survivors struggled to meet basic needs for themselves and their families, they began to reach out to DV organizations for assistance. A provider in California described this gap in resources, saying that “coronavirus has cut off so many peoples’ resources and necessities and money and food.” Another provider in Florida described clients reaching out for these essential resources saying, “I lost my job,” “My job dramatically reduced my hours,” or “I can’t pay my rent right now.” Several providers noted that these requests were not always solely from survivors currently experiencing domestic violence, and also came from former clients or even people just seeking resources. Amidst widespread financial pressure, housing insecurity, and family distress, simply needing basic resources may have contributed to survivors' decisions to seek support from DV organizations.

#### Stress and Close Confinement

In addition to the lack of material resources, almost every service provider reflected on an accelerated cycle of violence in relationships due to extended proximity and added stressors caused by the pandemic. They described survivors and abusers being forced into longer periods of close confinement, often without the short breaks of work or errands that would have typically occupied their time and provided moments of relief during the day. A provider from Vermont explained, “It’s more intense… a victim can say, ‘I can handle this for tonight,’ [but] now they have to handle it for 24 h, not just until dinner, or until the abuser falls asleep. It’s more concentrated and more time that they are exposed to and experiencing the same type of abuse.” Often, stress from this prolonged confinement was compounded by stressors brought on by the pandemic. As a provider in California noted:

There's no way for us to confirm, but we figured it’s because with survivors being trapped in their homes with their abusers, and the added stress of the financial uncertainty with losing food and jobs, the stresses of being together 24/7, we figured that contributed to their worries and insecurities, so they called more.

One of the most commonly discussed stressors was financial pressures brought on by the pandemic. One provider from Massachusetts described, “One difference I see, or it is getting worse, is clients reaching out because of financial instability. A lot of clients have lost their jobs, or their abuser lost their job, and their financial hardship has increased. They cannot pay their rent and are at risk of losing their homes.” Financial difficulties were discussed by providers in both urban and rural areas, servicing survivors from a variety of backgrounds. As added stress is a known risk factor for domestic violence (Cano & Vivian, 2001), an increase in psychological and economic stressors may help to explain the rise in contacts related to domestic violence.

#### Increased Lethality

Providers also associated this increase in stress with an increase in lethality of domestic violence cases, another explanation for the increase in contacts that organizations observed. A provider in California noted, “The other things that clients have been reporting to us is that the violence has increased due to stress in the family, the economic challenges, people lost their jobs... So the violence has definitely gone up, and it’s intensified.” A provider in Texas similarly emphasized the role of increased stress and close confinement as well as the lack of a de-escalation period:

It seems as if the cycle of violence in a household, the cycle is moving through it at a rapid rate than what it used to, and I think that’s because they don’t have a de-escalation time. Like when your partner goes to work, you would typically get de-escalation and then you could maybe enter back into the honeymoon phase when you’re home from work, right? They’re not getting that. So I’m seeing the same types of violence, I’m still seeing strangulation, still see guns, still see sexual assault, we see all of those things. It just seems that where maybe you would get strangled once a week, you’re now getting strangled every day.

An increase in sexual assault, strangulation, and weapons possession was also described by several other service providers. One provider from Georgia even recounted needing to create infographics about strangulation for the clients they serviced, “because we were seeing so much of that.” Given that survivors are more likely to reach out for services the more severe their cases become (Chang et al., 2010), an overall increase in the severity of cases may have contributed to the increase in DV reports described by interviewees.

### Changes in the Nature of DV

In addition to these increases and decreases in reported contacts, service providers also noted changes in the experiences of domestic violence survivors. For example, many interviewees shared accounts of abusers using the virus and health as an additional tool for control within the relationship. A provider from Louisiana described the experiences of survivors as “going beyond the usual isolation…not just I want you here, but now you *need* to be here.” One provider in Colorado recounted:

And we’ve also found some that have said, ‘I’ve had some symptoms or my child had some symptoms, and we were not allowed access to go to the doctor. They hid – my abuser hid our insurance cards, so I can’t go to the doctor.’ Just different things like that... Maybe a loved one has COVID and they’re not allowed to go and see them. They’re not giving them access to masks, so they can’t go anywhere. They’re not giving them access to hand sanitizers or disinfectants, so they’re not letting them protect themselves. A provider in Illinois shared a similar experience with a survivor, describing a case in which an abuser continued to bring people into the home during the lockdown, despite the survivor wishing to maintain social distancing. Providers also discussed how survivors had to navigate a lack of access to information, as well as deliberate misinformation, regarding COVID-19. Reflecting this concern, a provider from Missouri said, “A pandemic is another tool now that an abusive person can use to control another. Whether it be as simple as threatening to get the significant other sick on purpose, or using that fear and that control to further keep them from leaving. It’s another control tactic to immobilize and keep somebody in fear of their life.”

Another common theme in service provider interviews was a newfound lack of social support among survivors and an increase in isolation. For example, a provider from California described, “They're not coming into contact with as many people, so they're less likely to have disclosed to someone else, they're less likely to have been encouraged by someone else. They're more isolated, and being isolated is already one of the factors for DV anyway.” In addition to the role that social ties can play in helping survivors access resources, survivors also struggled with simply not having people to confide in. A provider in Minnesota expanded on how this affected survivors’ coping abilities:

I think people’s emotional responses to the abuse they’re experiencing is a lot worse now…is a lot deeper, it cuts deeper on the people who are experiencing it, because... the community support services are gone, they don’t have the ability to go and talk to your girlfriends and decompress... people are increasingly isolated and having to deal with the abuse they’re experiencing...and so the emotional turmoil, the emotional effect of the abuse has been a lot more severe.

This newfound lack of social support was especially prominent in interviews with those who specifically worked with survivors from marginalized communities. For example, a provider from New York stressed the detrimental effect that the closing of community spaces had on the immigrant LGBTQ + survivors with whom they were working. They detailed:

I think also because most of my clients are immigrants, queer or trans folks… So much of immigrant, especially recent immigrant, queer/trans community is community spaces, and with the absence of those, people have become way more isolated… especially since most of my clients are disconnected from their families due to homophobia and transphobia. And normally community spaces take the place of that, but now it’s more challenging than usual.

A provider working with disabled survivors in New York noted, “I think our population’s needs really differ from other populations, just working with people with disabilities and who are deaf. The isolation and the communication and the barriers, they’re just unique to our population… our population is normally isolated, now they’re just further isolated.” With fewer opportunities for in-person interaction for most people in society, those most vulnerable may have been particularly negatively impacted.

## Discussion

Our results highlight the unique challenges faced by domestic violence service providers and the survivors that they supported during lockdown across the United States. As the risk of domestic violence escalated during stay-at-home periods, service providers put in great effort to provide accessible and holistic services, help survivors navigate new stressors, and combat misinformation. The shelter-in-place mandates introduced during the COVID-19 crisis further weakened the existing fragmented resource infrastructure for survivors, accelerated the cycle of violence that many survivors remained caught in, and exacerbated barriers to accessing help.

As scholars and policymakers work to interpret quantitative descriptions of trends in domestic violence during the pandemic, these findings provide insight into the lived experiences driving both the decreases and increases in contact volume. Importantly, our findings reveal that survivors not reaching out during a time of crisis does not reflect lack of violence, but rather new obstacles to help-seeking. We also build upon existing literature on experiences of DV during disasters (e.g., Serrata & Hurtado Alvarado, 2019; Lauve-Moon & Ferreira, 2017; Buttell & Carney, 2009; Schumacher et al., 2010) by identifying some of the specific barriers that emerged as a result of the pandemic, including how the virus itself was used as a tool of control and the multi-dimensional isolation created by stay-at-home orders. Still, these findings are valuable to keep in mind when considering domestic violence in general, as common underlying factors such as competing survival needs, lack of clarity around accessible resources, and economic stressors will likely remain significant barriers for survivors long after the COVID-19 pandemic (Sety et al., 2014).

Insight into the challenges that survivors faced points to potential ways that governments and public health officials can better respond to survivors’ unique needs during times of crisis (Chandan et al., 2020). As the COVID-19 pandemic evolves, leaders from state and local governments, DV service organizations, schools, first responders, and health providers must come together to understand interrelated vulnerabilities that survivors and their families face, mobilize resources to meet survivors’ unique needs, and develop coordinated responses to better support survivors and their families (Campbell, 2020; Kofman & Garfin, 2020). Diverse stakeholder engagement and service coordination are paramount to effectively identifying and assisting at-risk individuals, especially during shelter-in-place periods that leave many survivors with few community touchpoints. For example, stakeholders can partner with other “essential organizations,” such as pharmacies and grocery stores, to safely provide more public information about DV resources. Such partnerships could potentially integrate discrete reporting platforms to provide survivors with more avenues to access resources.

Our study also finds that survivors were unclear as to what resources were available during lockdown, which prevented them from accessing necessary help. In order to effectively support those in need, a multifaceted approach to reaching survivors is necessary. In addition to providing discrete information about DV resources through multiple digital platforms and in-person touchpoints, DV organizations can cultivate partnerships for coordinated responses with community service providers such as organizations that provide housing, food, child care, health care, and government services, allowing information to spread through multiple kinds of services. On a broader scale, because mobilizing a quick response to new barriers requires substantial resources, local and state governments must adequately support domestic violence organizations in relief efforts not only during the peak of a crisis, but also in the months and years to come.

This study is not without its limitations. Because there was no national shelter-in-place mandate given by the U.S. federal government, shelter-in-place orders varied across different states, cities, and counties, with some being more lenient (i.e., keeping schools and non-essential businesses open) and others being more strict. Thus, the pandemic and its resulting shelter-in-place orders may have affected survivors in different areas differently, perhaps accounting for some variation seen in our results. We were unable to analyze the potential effects of differences among mandates in this study, although this would be an interesting avenue for future research. Future studies may also want to sample providers from cities and states not included in this analysis to ensure transferability of these results. Additionally, some cities and states had multiple provider interviews while others had only one. This may have allowed a trend seen in one particular region to dominate in the analysis, although we tried to ensure that any overall trends presented here were seen across a majority of interviews.

Furthermore, it is important to recognize that not all survivors of domestic violence make contact with DV service organizations. While speaking to survivors themselves would have provided valuable insight, they were not a realistic study population for this research. Survivors currently experiencing domestic violence are an extremely vulnerable, hard-to-reach group whose main priority is remaining safe and alive. Moreover, depending on the timing of abuse, survivors may not be able to address changes in their experiences from pre-pandemic to the pandemic period, which is one of the main aims of this research project. Beyond the limitations of interviewing providers more broadly, the composition of the sample may have also shaped our findings. Our sample was predominantly female, and most worked at organizations that primarily supported female survivors. As a result, the experiences of non-female survivors, who may face unique obstacles of their own to accessing support, may be underrepresented in this data.

This study adds to our understanding of the impact of the COVID-19 pandemic on domestic violence in the United States by identifying some of the key factors influencing survivors’ experiences of DV and ability to access support in the months during and following the first wave of shelter-in-place orders. However, additional empirical research on this topic, both qualitative and quantitative, is crucial. One of the unexpected trends seen in many of our interviews was service providers detailing various program adaptations made by their organizations (e.g., developing a chat or text feature on their website, services provided over video chat). Future research may want to further explore these adaptations and study their long-term effectiveness. Given that additional waves of COVID-19 infections and corresponding shelter-in-place mandates have occurred in many countries, researchers may want to investigate the impact of later shelter-in-place mandates on survivors of domestic violence, perhaps comparing experiences at multiple time points.

## Conclusion

In response to the COVID-19 pandemic, “stay at home” became the mantra of governments and public health organizations. Unfortunately, results from this study suggest that for those experiencing domestic violence, home was not a place of safety during this time. At the start of stay-at-home orders, providers found that survivors of domestic violence struggled to reach out for help as they faced numerous new and exacerbated barriers, such as greater safety concerns, competing priorities, and a lack of clarity surrounding what resources were available. Many organizations faced increased demand after shelter-in-place orders were lifted and survivors were able to connect to support. As they served clients during periods of greater demand, providers noted consistent factors influencing survivors’ experiences of domestic violence, including a lack of basic resources, changes in the cycle and lethality of violence, the use of the virus itself as a tool of control, and greater isolation from necessary social support. These findings provide context for previously-observed changes in rates of domestic violence by describing the factors underlying these changes. Better understanding these factors can shape future public health responses, allowing for interventions that both keep survivors safe during times of crisis and work towards a “new normal” where everyone is able to return to a safe home.
